# Integrative analysis workflow for the structural and functional classification of C-type lectins

**DOI:** 10.1186/1471-2105-12-S14-S5

**Published:** 2011-12-14

**Authors:** Geoffrey Koh, Ariana Low, Daren Poh, Yujian Yao, Say Kong Ng, Victor Vai Tak Wong, Vincent Vagenende, Kong-Peng Lam, Dong-Yup Lee

**Affiliations:** 1 Bioprocessing Technology Institute, Agency for Science, Technology and Research (A*STAR), 20 Biopolis Way, #06-01, Centros, 138668, Singapore; 2 Department of Chemical and Biomolecular Engineering, National University of Singapore, 4 Engineering Drive 4, 117576, Singapore

## Abstract

**Background:**

It is important to understand the roles of C-type lectins in the immune system due to their ubiquity and diverse range of functions in animal cells. It has been observed that currently confirmed C-type lectins share a highly conserved domain known as the C-type carbohydrate recognition domain (CRD). Using the sequence profile of the CRD, an increasing number of putative C-type lectins have been identified. Hence, it is highly needed to develop a systematic framework that enables us to elucidate their carbohydrate (glycan) recognition function, and discover their physiological and pathological roles.

**Results:**

Presented herein is an integrated workflow for characterizing the sequence and structural features of novel C-type lectins. Our workflow utilizes web-based queries and available software suites to annotate features that can be found on the C-type lectin, given its amino acid sequence. At the same time, it incorporates modeling and analysis of glycans - a major class of ligands that interact with C-type lectins. Thereafter, the results are analyzed together with context-specific knowledge to filter off unlikely predictions. This allows researchers to design their subsequent experiments to confirm the functions of the C-type lectins in a systematic manner.

**Conclusions:**

The efficacy and usefulness of our proposed immunoinformatics workflow was demonstrated by applying our integrated workflow to a novel C-type lectin -CLEC17A - and we report some of its possible functions that warrants further validation through wet-lab experiments.

## Background

C-type lectins are Ca^2+^-depending sugar-binding proteins that are involved in several immune-related and other physiological functions. They are ubiquitous in the animal kingdom, and exist mostly as membrane receptors. Indeed, C-type lectins play an important role in pathogen recognition and cell-cell interaction through specific binding with glycans (sugars) found on the surfaces of target cells and glycosylated molecules [1]. The importance of understanding C-type lectins and finding their interacting partners (both glycans as well as other molecules) is exemplified by applications in immuno- and vaccination-therapies, where lectins expressed on cells such as Dendritic cells (DCs) can be targeted by their natural ligands or antibodies that are directed against them. Such ligands are usually conjugated with antigens, which can be presented to T-cells upon ligand binding, leading to subsequent T-cell maturation and development of immunity towards the antigen [2]. C-type lectins also have extensive applications in protein engineering, where mutations can be made to specific sites to modify their specificity towards certain ligands. Such modifications can be made only when we have a better understanding of their structural and functional characteristics [3].

Presently, 17 groups within the C-type lectin superfamily have been recognized [4], with more C-type lectins being constantly discovered based on the presence of a conserved 115-130 amino acid domain along their sequences - the C-type *carbohydrate recognition domain* (CRD). However, for most of the recently identified C-type lectins, their interactions with carbohydrates, intracellular functions and molecular mechanisms still remain unclear. Thus it is highly needed to characterize these proteins in order to uncover their possible physiological and pathological roles in the immune system. On a similar note, it is also imperative to develop techniques in glycoinformatics, so as to aid the elucidation and analysis of protein-glycan interactions - one of the key processes in the mammalian immune system [5].

To this end, we propose an integrative analysis workflow that utilizes various techniques and algorithms to systematically discover and annotate the putative functions of novel C-type lectins. Our workflow starts with the amino acid sequences to predict the primary functional units, i.e. domains and motifs. It is followed by homology modeling to determine the molecular structures of the C-type lectins. In tandem with this step is the generation of glycan conformer libraries, with the glycan composition being obtained from various sources and possibly specified in different formats. Finally, computational virtual screening is performed to identify potential protein-glycan interactions.

## Methods

### Integrative workflow for sequence and functional analysis of C-type lectins

It is possible to predict the putative functions of novel C-type lectins by analyzing their amino acid sequences and structures. This is due to the accepted view that protein functions can be ‘*inherited through homology*’ [6]. In general, a peptide is composed of independently functioning smaller units, i.e. domains. Together with the advent of computational methods to identify these domains along a protein sequence, and the growing collection of known domains and their associated functions, e.g. Pfam [7], PROSITE [8], SMART [9], and InterProScan [10], it becomes evident that the first steps to analyze an unknown C-type lectin is to search its sequence for conserved domains. These domains indicate the possible functions, interactions and cellular locations of the C-type lectin, and also the secondary and tertiary structures it may assume.

Aside from sequence-based analysis, one can also study C-type lectins through their molecular structures, which can be either obtained through computational prediction [11], or determined by x-ray crystallography. Such *physicochemical* approaches can aid in understanding the molecular mechanisms of their functions at the atomic level. For instance, van Liempt *et al*. [12] analyzed the molecular structures of the C-type lectins DC-SIGN and L-SIGN, and identified the residues that were responsible for the differences in their carbohydrate binding profiles. Glazer *et al*. [13] further improved the prediction of potential Ca^2+^ binding sites by incorporating molecular dynamics to the protein structures. Going forward, docking studies and *in silico* screening can be performed against virtual libraries of glycans [14]. This is already an integral part of the industrial drug discovery process for other proteins [15].

**Figure 1:**
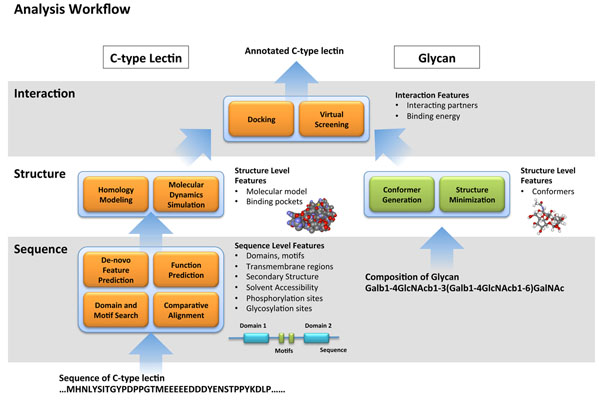
**Analysis workflow.** A schematic illustration of the integrated workflow. The left side of the panel summarizes the steps for the sequence and structural characterization of novel C-type lectins at various levels. The right side shows equivalent analyses for glycans that is needed in order to construct a virtual library amenable for virtual screening.

Herein, we proposed an analysis workflow where the various approaches for predicting the structures and functions of proteins are systematically integrated to characterize a novel C-type lectin, given its sequence information. Figure 1 illustrates the schematic workflow, which operates in a bottom-up manner, starting from sequence-based analysis, and subsequently predicting the molecular structure. Parallel to this step is the generation of conformers (molecular structures) for glycans based on the identity of their monosaccharide subunits and linkages. Finally the C-type lectin model can then be screened against the in silico glycan library to elucidate possible interactions.

### Sequence-based analysis

There is a plethora of different sequence analysis algorithms that can identify domains and motifs within a protein sequence. For instance, PROSITE scans a query protein sequence against an internal database of sequence signature patterns which were curated from literature. In addition, for each pattern, there is a *miniprofile* to refine the hits, as well as post-processing of the matches with some contextual information to improve accuracy [8]. On the other hand, Pfam stores its database of protein domains as hidden Markov models (HMMs) and uses the HMMER3 algorithm to determine the presence of the domains within a query protein sequence [7]. As such, the first step for analysis will be to leverage these existing platforms in order to gather as much information as possible, given a C-type lectin amino acid sequence.

Most of the domain/motif prediction algorithms have been implemented and their services are accessible through form-based interfaces over any web browsers. Table 1 shows a non-exhaustive list of available algorithms for sequence-based analyses on the given C-type lectin sequences. Thus we have prototyped an in-housed web-based interface to automate the querying of the various servers, e.g. Pfam, SMART, via hypertext transfer protocol (HTTP) requests, thereby allowing us to quickly access various sequence-based algorithms using their most updated profile databases. Details of how the queries are sent and the results are visualized can be found in Additional File 1. It should also be noted that by delegating the analyses of C-type lectin sequences to the various web servers, downloading and installing their prediction programs locally, e.g. NetOGlyc 3.1 [16] and NetNGlyc 1.0, become optional, thus alleviating some of the issues caused by incompatible operating systems or shell environments.

**Table 1:** List of servers and algorithms

SN	Server (URL)	Type of features
1	Pfam (http://pfam.sanger.ac.uk)	Domains
2	Prosite (http://expasy.org/prosite)	Domains, motifs
3	SMART (http://smart.embl-heidelberg.de)	Domains, motifs
4	TMHMM 2.0 (http://www.cbs.dtu.dk/services/TMHMM)	Transmembrane helix
5	NetNGlyc (http://www.cbs.dtu.dk/services/NetNGlyc)	N-linked Glycosylation
6	NetOGlyc (http://www.cbs.dtu.dk/services/NetOGlyc)	O-linked Glycosylation
7	Phospho.ELM (http://phospho.elm.eu.org)	Phosphorylation Sites
8	ELM (http://elm.eu.org)	Eukaryotic linear motifs

The table shows a non-exhaustive list of web servers that can be queried to predict various sequence-based features.

### Molecular modeling

The next step in our workflow is to construct the molecular structure of the C-type lectin. Here, homology modeling can be employed to predict its structure. Generally, homology modeling of C-type lectins follows a series of steps - (i) template selection, (ii) structural alignment, (iii) model construction and constraint satisfaction, and (iv) refinement. For template selection, the sequence of the C-type lectin is first queried against the set of non-redundant proteins in the PDB database using the BLASTp algorithm [17]. Proteins with moderate levels of sequence identity, typically more than 30% of the aligned regions [18], are then chosen as templates for modeling.

Note that there can be multiple templates, especially when they are aligned to different regions of the query protein. In addition, it is not always the case where the entire C-type lectin can be modeled. As the CRD is the most highly conserved region of C-type lectins, its homologs can usually be found in the PDB database. Upon selection of the templates, the query sequence and the templates are re-aligned based on a more stringent set of criteria which include fractional side chain accessibility and secondary structure type. Finally, using the template structures, the model is constructed by initially copying the coordinates of the backbone atoms (C, Cα, N and O) of aligned residues. It is followed by filling the gaps (i.e. loop and gap modeling), adding side chain residues to the backbone amino acids, and adjusting the model to make sure that spatial constraints are not violated [19]. Depending on the level of alignment between the query C-type lectin and template sequences, an additional refinement step via molecular dynamics simulation may be required. In our workflow, all four steps are performed using the software suite Discovery Studio 2.5 by Accelrys, Inc [20]. This part of the workflow is not yet automated due to the manual intervention for the selection of templates during the model construction. There are, however, some existing works that have attempted to simplify molecular modeling into a one-step process [21,22] and these may be incorporated into our workflow later on.

As there is no crystal structure available for most of the novel C-type lectins, the predicted structures can only be validated using algorithms that assess their correctness based on physicochemical properties such as planarity, chirality and bond length deviations [23] of the residues. PROCHECK [24] is one of the software packages performing this function. In our case, we use the Profiles-3D methology [25] for structure validation. In addition, for each structure being constructed, its Ramachandran diagram is also plotted and analyzed to detect significant violations of the psi-phi angles between the amino acid residues [26]. We select the best scoring model that has no gross physicochemical violations for further analysis and classification. Having obtained the molecular model of the C-type lectins, we can then perform docking studies to identify their putative binding partners.

### Glycan conformer generation

For docking simulations, the structures of both the receptors and ligands must be known. In our current setting, C-type lectins are the receptors for glycan molecules. Having obtained their structures through homology modeling, we now require the glycan structures. Despite the availability of small ligand databases such as ZINC [27], they are not specific to glycans, thus making it difficult to search for the relevant models. Moreover, with the huge diversity of natural and synthetic glycans, it is technically challenging to resolve their structures and store them in databases.

**Figure 2:**
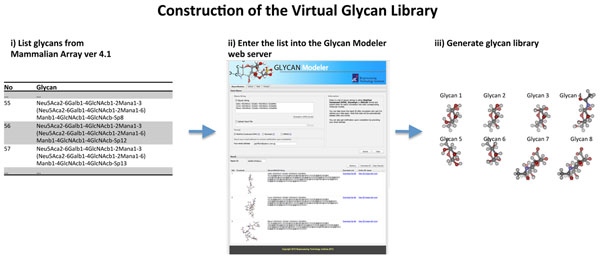
**Construction of in silico library**. The glycans from the array are listed out (in the modified condensed IUPAC format) and submitted to our glycan modeling server for generating conformers that are amenable for docking studies.

For this part within the workflow, we have developed an alternative approach. Instead of storing known glycan structures, we generate them ‘on-the-fly’. Starting from a linear representation of the glycan structures (in either the modified condensed IUPAC or Glycodigit [28] formats), we rewrite them into a more generic form -SMILES (simplified molecular input line entry specification) [29] - and utilize readily available software (Balloon [30]) to generate the different structures amenable for docking studies. We have implemented this process as a web-based application and it is available at the link (http://bioinfo1.bti.a-star.edu.sg/glycan/). Following the approach (as summarized in Figure 2), we constructed an *in silico* library on the basis of the glycan arrays developed by the Consortium of Functional Glycomics [31,32]. Currently we have 509 structures out of the 511 glycans on the glycan array with a coverage of 99.6%.

### Virtual screening

The final step in the functional classification of C-type lectins in our workflow is to screen for plausible interactions with the glycan library through computational docking studies. We use LigandFit, an algorithm that locates possible binding sites by analyzing cavities in the protein structure before trying to dock each glycan from our virtual library [33]. The output from this virtual screening is a list of glycans that have plausible poses in any of the predicted binding sites.

## Results and discussion

### Sequence Analysis of CLEC17A

**Figure 3:**
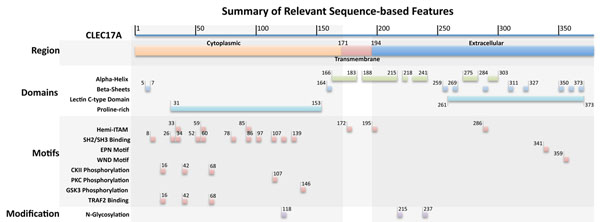
**Features of CLEC17A**. Summary of the relevant features found along the sequence of CLEC17A.

We applied our workflow on CLEC17A [Uniprot: Q6ZS10], a receptor that is expressed on dividing B cells in germinal centers [34]. CLEC17A was first identified and given the symbol by the HUGO Gene Nomenclature Committee. However, much remains to be done to elucidate its function and role in the immune system. Here we attempt to add to the knowledge on CLEC17A by running its amino acid sequence through our analysis workflow. The relevant sequence-based features are summarized in Figure 3. The full list of predicted features is provided in Additional file 2.

From the results, CLEC17A is a Type II transmembrane protein. As a C-type lectin, it is predicted to have a high specificity towards mannose and Ca^2+^ due to the presence of the EPN motif (position 341) and WND motif (position 359) respectively. Within the extracellular region, there are two predicted N-linked glycosylated sites (positions 215 and 237), which may play a physiological role in the transport and localization of CLEC17A to the cell surface [35]. We used some of these results to complement the experimental investigation and analysis of N-linked glycosylation sites on CLEC17A (See Additional File 3)

For the cytoplasmic region, there are several domains and motifs of interest. In particular, a number of SH2 and SH3 recognition domains can be found within a proline-rich region. The same SH2 binding motifs are also predicted to be phosphorylated by proline-directed kinases. A possible candidate would be the mitogen-activated protein kinase (MAPK). This adds to the confidence that SH2 containing proteins such as the adaptor protein Grb2 and Src family proteins can dock to the cytoplasmic tail of CLEC17A. Another possible intracellular signaling mechanism can be inferred by the presence of hemi-ITAM motifs (YxxL). This motif, which is also present in Dectin-1, can recruit and activate the Syk family kinases [36]. Incidentally, Syk also has SH2 domains, supporting the hypothesis that it interacts with CLEC17A.

Casein kinase II (CKII) is predicted to be another kinase that may phosphorylate CLEC17A based on its recognition motif ([ST]xx[DE]). Following the consensus between Prosite and ELM, the possible phosphorylation sites were shortlisted to positions 16, 42, and 68. Furthermore, these regions are enriched with glutamic acid, providing the acidic context for CKII phosphorylation [37]. Other potential kinases for CLEC17A include protein kinase C (PKC) at position 107 and glycogen synthase kinase-3 (GSK3) at position 146, the latter being less reliable as the specificity of GSK3 has not been confirmed. Of note is the presence of TNF receptor-associated factor 2 (TRAF2) binding motif ([PSAT]x[QE]E) [38]. Although TRAF2 is commonly associated with the tumor necrosis factor receptor (TNFR) superfamily, it has been suggested by Geijtenbeek and Gringhuis [39] that the activation of nuclear factor NF-κB by Dectin-1 may involve the recruitment and activation of TRAF2-TRAF6 complex. Since there are some similarities in the cytoplasmic motifs found in Dectin-1 and CLEC17A, it is possible that this interaction is present in CLEC17A intracellular signaling as well. Nevertheless, confirmation of these features awaits experimental verification.

There are several other regulatory motifs that were found by the prediction servers. However, the biological context for their functions were not present in CLEC17A, and hence were not considered further. For instance, the C-terminal binding protein (CtBP) interacting motif (position 121) occurs mostly in DNA-interacting proteins and transcription factors. Since CLEC17A is a transmembrane receptor, this motif is discarded as a false positive.

### Structure prediction and docking studies of CLEC17A

**Figure 4:**
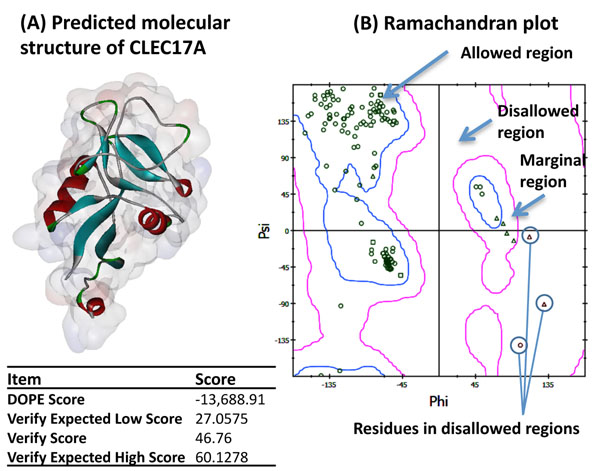
**Predicted structure of CLEC17A.** (A) Homology modeling of CLEC17A and the score of its structure calculated by Profiles-3D. (B) Ramachandran plot of the psi-phi angles between all amino acid residues of the predicted. Most of the residues fall within allowed regions (95.7%), a small percentage of residues are within the marginal regions (3.4%), and only 3 residues are located in the disallowed region (0.9%).

The molecular structure of CLEC17A was predicted by comparative homology modeling using the following proteins as templates - (i) CD209 antigen-like protein 1 [PDB Id: 1XPH], (ii) Collectin placenta 1 [PDB Id: 2OX9], and (iii) mDC-SIGN1B Type I isoform [PDB Id: 1SL4]. However, these templates can only be aligned to the CRD domain of CLEC17A (from 194 to 378) and hence the structure can only be constructed within this region. The sequence identity and similarity of the CRD between CLEC17A and its template sequences was 29.7% and 53.1% respectively. The final model was created using the MODELLER algorithm [19]. Five models were created, and they were sorted by probability density function (PDF) total energy scores. Thereafter the model with the lowest score was chosen, and its loop regions were further refined using MODELLER’s DOPE-based loop modeling protocol [40]. The final structure is depicted in Figure 4A. The predicted result was validated by Profiles-3D [25], showing that the model structure is acceptable based on the verify scores. The Ramachandran diagram was also plotted to determine the proportion of residues that violate the psi-phi angle constraints (Figure 4B). Most residues are within allowable or marginal regions, while only a few (0.9%) fall within the disallowed region, indicating a high level of correctness for the structure.

**Figure 5:**
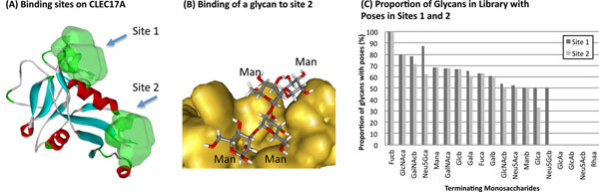
**Virtual screening of CLEC17A against the in silico glycan library.** (A) Binding sites on CLEC17A that were screened against the glycan library. (B) Structure of a glycan terminating with mannose bound to site 2 on CLEC17A. (C) Proportion of the glycans in the library terminating with the respective monosaccharides, and having plausible poses on binding sites 1 and 2.

We analyzed the cavities on the surface of the CLEC17A model, resulting in four putative binding sites, two of which can be considered for virtual screening against the in silico glycan library (Figure 5A). The other two sites were deemed improbable as they are solvent inaccessible cavities. To further validate our assumption, we docked the structures of mannose-α and fucose-α to the four binding sites using the LibDock protocol [41]. Of the four sites, only the two surface binding sites returned plausible solutions (poses).

Next, we moved on to the virtual screening of the two surface binding sites against the glycan library using the following docking protocols - (i) CDocker, (ii) LibDock and (iii) LigandFit. In order to render the poses from the different protocols comparable, we re-scored them using a set of standard scoring functions -LigScore1,2 [42], Piecewise linear potential (PLP1,2) [43], Jain [44], and potential of mean force (PMF) [45]. A consensus score is then generated for each ligand. Finally, the ligand poses are sorted according to the consensus score, and the top 25% unique ligands for each binding site are selected for further analysis.

As an initial analysis of the global glycan binding profile of CLEC17A, we looked at the terminating monosaccharides of the dockable glycans: it has been suggested in Taylor and Drickamer [46] that the binding specificities of C-type lectins may be due to their interaction with the terminal sugar. Hence, for each type of terminal monosaccharide, we obtained the list of corresponding glycans from the library and computed the proportion that docks to CLEC17A (Figure 5C). The results suggested that CLEC17A, in addition to its specificity towards mannose, may also bind glycans terminating with sugars such as fucose-β, N-glycolylneuraminic acid-α, N-acetylglucosamine-α and N-acetylgalactosamine-β. Note that as this is an initial analysis, a more thorough approach might be required to confirm the possible interactions between CLEC17A and the glycans, as well as the amino acid residues responsible for forming the bonds.

## Conclusions

In this work, we have collected various methods for analyzing the putative structures and functions of novel C-type lectins and incorporated some of them into an integrative workflow for studying such lectins in a bottom-up manner. Sequence-based motifs and domains are first identified using an integrative metaserver. The structure of the given lectin is then constructed by homology modeling, and its putative functions are assessed through virtual screening against an *in silico* library of glycans that are found in mammalian cells. Having such a workflow in place will significantly increase the speed and efficiency of identifying the putative roles and functions of novel C-type lectins for further experimental validation. We applied our workflow to elucidate the putative functions of a novel human C-type lectin -CLEC17A, and characterized it as a N-linked glycosylated transmembrane protein with high specificity towards mannose and fucose. Preliminary screening studies have also shown that CLEC17A possibly binds glycans that terminate with a few other monosaccharides such as N-glycolylneuraminic acid and N-acetylglucosamine. Additionally, the presence of motifs that bind to SH2 and SH3 domains, as well as the hemi-ITAM motifs suggests that CLEC17A is involved in intracellular signaling which could lead to the production of cytokines such as interleukins.

With the development of more algorithms to predict sequence and structural features on C-type lectins, several more possible cellular functions of lectins may be revealed. However, the algorithms will have varying sensitivity and specificity. Although not all of them have been integrated into the workflow yet, we have demonstrated that integrating and interpreting the results together are invaluable in both filtering out improbable predictions and aiding the design of future experiments for validation. With all the collated results, future work will include probabilistic approaches for accepting or rejecting prediction results.

Moreover, some parts of our workflow still require human supervision. At present, there are some works that aim to achieve the complete automation of homology modeling [21,22], and these can be integrated within our workflow to make it as an entirely automated process in the future. Incorporating the workflow with systems-level analysis such as pathway information will also shed more light not only on the features of the novel C-type lectins, but also their molecular mechanisms and functions from a network-centric point of view. In addition, we are currently developing an in-house database system to store information on C-type lectins and their interacting partners, and it will be designed to allow direct entry of information from the prediction results generated via the workflow.

## Competing interests

The authors declare that they have no competing interests.

## Authors’ contributions

GK and LDY conceived the idea, and wrote the manuscript. DP performed some of the sequenced based analysis in this work. AL worked on the computational docking simulations, and together with NSK, they performed the experimental investigations into the N-glycosylation sites on CLEC17A. GK and YY worked on creating the glycan modeling module. VVTW, VV, NSK and LKP provided ideas towards the development of the workflow. They also participated in the analysis and intepretation of the prediction results on CLEC17A, as well as the proof-reading of the manuscript.

## Supplementary Material

Additional file 1 XML schema definition (XSD) for the query results. Click here for file

Additional file 2 The full list of predicted domains and motifs on CLEC17A. Click here for file

Additional file 3 Additional background, as well as materials and methods for the experimental investigation of predicted N-glycosylation sites. Click here for file
