# Supplementary material for: Integrative analysis workflow for the structural and functional classification of C-type lectins
Source: BMC Bioinformatics. 2011 Dec 14;12(Suppl 14):S5. doi: 10.1186/1471-2105-12-S14-S5 (PMC3287470; doi:10.1186/1471-2105-12-S14-S5)
Supplement: Additional file 1 — XML schema definition (XSD) for the query results. [file 1471-2105-12-S14-S5-S1.doc]

# Additional File 1

**Workflow Implementation**

The prototype of the workflow is implemented using a browser-based interface coupled with AJAX queries to the individual web servers, as shown in the figure below.


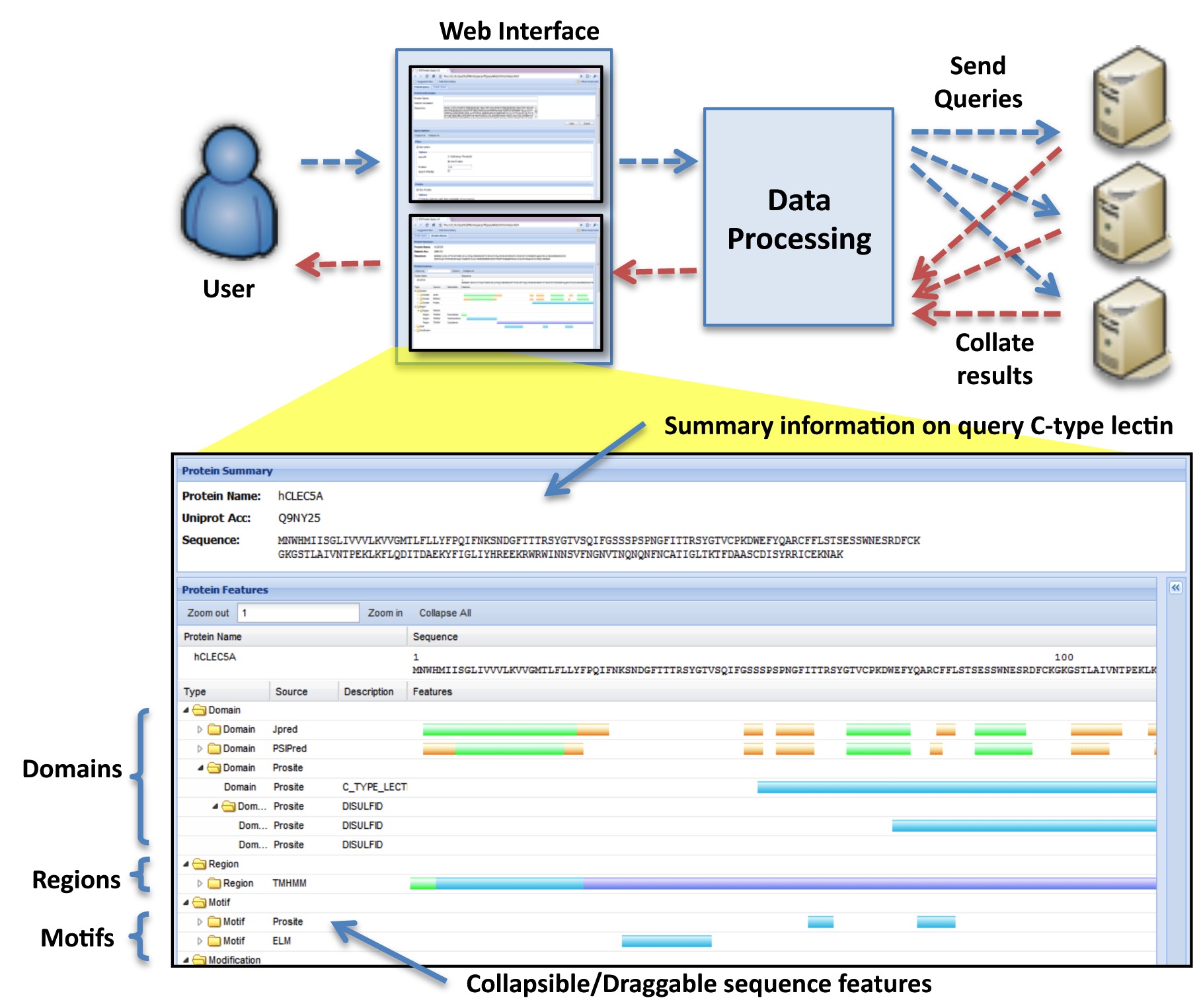


**Figure 1 - Querying and result visualization** Prototype interface for querying the various sequence analysis servers and visualizing the results. Users can submit lectin sequences through a web-based interface, which will then send them to various prediction servers. Results are then collated and displayed to the user for interpretation and analysis. The domains and motifs are grouped and displayed as collapsible entries to allow users to choose their desired level of details.

For each server, we have a query object to send the C-type lectin sequence as a HTTP request. Since the individual calls are asynchronous, the workflow will not break in the event any of the servers is unreachable (for maintenance and the such). A timeout mechanism is set for each query object such that it does not perpetually wait for a response.

## Result Visualization

Upon querying against the various servers and algorithms, the results need to be visualized in an integrated manner for easy interpretation and comparison. To this end, the different result formats are each parsed into a standard XML representation (See Listing 1) and displayed via an interactive interface. In order to reduce clutter, we proposed to classify the different features into the following categories - (i) region (ii) domain (iii) motif and (iv) modification. Within each category, similar features (e.g. N-linked glycosylation sites) should be grouped together for ease of analysis. By integrating and aligning the results together, the features can be placed in their proper biological context for analysis and subsequent validation. For example, the presence of N-linked glycosylation can be detected by locating the Asn-Xaa-Ser/Thr/Cys motif (where Xaa is any amino acid except Proline) along the amino acid sequence of a C-type lectin. However, since C-type lectins are mostly transmembrane proteins, the motifs that fall within the cytoplasmic region can be disregarded as false positives. Currently, this validation process is still dependent on prior biological knowledge of the researchers. Nonetheless, availability of these results will allow a more systematic approach for validation to be developed.

| <?xml version="1.0" encoding="UTF-8"?>  <xsd:schema elementFormDefault="qualified" xmlns:xsd="http://www.w3.org/2001/XMLSchema">  <xsd:element name="Protein" type="ProteinType"></xsd:element>  <xsd:complexType name="ProteinType">  <xsd:sequence>  <xsd:element name="FeatureArray" type="FeatureArrayType" maxOccurs="1"  minOccurs="0"></xsd:element>  <xsd:element name="AnnotationArray" type="AnnotationArrayType" maxOccurs="1"  minOccurs="0"></xsd:element>  </xsd:sequence>  <xsd:attribute name="proteinShortName" type="xsd:string"></xsd:attribute>  <xsd:attribute name="proteinLongName" type="xsd:string"></xsd:attribute>  <xsd:attribute name="proteinAlternateNames" type="xsd:string"></xsd:attribute>  <xsd:attribute name="uniprotID" type="xsd:string"></xsd:attribute>  <xsd:attribute name="uniprotURL" type="xsd:anyURI"></xsd:attribute>  <xsd:attribute name="isoform" type="xsd:int"></xsd:attribute>  <xsd:attribute name="sequence" type="xsd:string" use="required"></xsd:attribute>  <xsd:attribute name="sequenceLength" type="xsd:string" use="required"></xsd:attribute>  <xsd:attribute name="organism" type="xsd:string"></xsd:attribute>  </xsd:complexType>  <xsd:complexType name="ProteinAnnotationType">  <xsd:sequence></xsd:sequence>  <xsd:attribute name="category" type="xsd:string"></xsd:attribute>  <xsd:attribute name="annotation" type="xsd:string"></xsd:attribute>  <xsd:attribute name="description" type="xsd:string"></xsd:attribute>  <xsd:attribute name="sourceType" type="xsd:string"></xsd:attribute>  <xsd:attribute name="source" type="xsd:string"></xsd:attribute>  <xsd:attribute name="sourceID" type="xsd:string"></xsd:attribute>  <xsd:attribute name="significance">  <xsd:simpleType>  <xsd:restriction base="xsd:float">  <xsd:minInclusive value="0"></xsd:minInclusive>  <xsd:maxInclusive value="1"></xsd:maxInclusive>  </xsd:restriction>  </xsd:simpleType>  </xsd:attribute>  <xsd:attribute name="score" type="xsd:double"></xsd:attribute>  <xsd:attribute name="scoreDescription" type="xsd:string"></xsd:attribute>  </xsd:complexType>  <xsd:complexType name="ProteinFeatureType">  <xsd:attribute name="featureType" type="xsd:string"></xsd:attribute>  <xsd:attribute name="featureDescription" type="xsd:string"></xsd:attribute>  <xsd:attribute name="feature" type="xsd:string"></xsd:attribute>  <xsd:attribute name="featureVisible" type="xsd:boolean"></xsd:attribute>  <xsd:attribute name="start">  <xsd:simpleType>  <xsd:restriction base="xsd:int">  <xsd:minInclusive value="1"></xsd:minInclusive>  </xsd:restriction>  </xsd:simpleType>  </xsd:attribute>  <xsd:attribute name="end">  <xsd:simpleType>  <xsd:restriction base="xsd:int">  <xsd:minInclusive value="1"></xsd:minInclusive>  </xsd:restriction>  </xsd:simpleType>  </xsd:attribute>  <xsd:attribute name="sourceType" type="xsd:string"></xsd:attribute>  <xsd:attribute name="source" type="xsd:string"></xsd:attribute>  <xsd:attribute name="sourceID" type="xsd:string"></xsd:attribute>  <xsd:attribute name="sourceURL" type="xsd:anyURI"></xsd:attribute>  <xsd:attribute name="significance">  <xsd:simpleType>  <xsd:restriction base="xsd:float">  <xsd:maxInclusive value="1"></xsd:maxInclusive>  <xsd:minInclusive value="0"></xsd:minInclusive>  </xsd:restriction>  </xsd:simpleType>  </xsd:attribute>  <xsd:attribute name="score" type="xsd:double"></xsd:attribute>  <xsd:attribute name="scoreDescription" type="xsd:string"></xsd:attribute>  </xsd:complexType>  <xsd:complexType name="FeatureArrayType">  <xsd:sequence>  <xsd:element name="ProteinFeature" type="ProteinFeatureType"  maxOccurs="unbounded" minOccurs="0"></xsd:element>  </xsd:sequence>  </xsd:complexType>    <xsd:complexType name="AnnotationArrayType">  <xsd:sequence>  <xsd:element name="ProteinAnnotation" type="ProteinAnnotationType"  maxOccurs="unbounded" minOccurs="0"></xsd:element>  </xsd:sequence>  </xsd:complexType>  </xsd:schema> |
| --- |

**Listing 1 - XML Schema** The XML schema to represent the pertinent information for the results obtained from different servers
