# Supplementary material for: Integrative analysis workflow for the structural and functional classification of C-type lectins
Source: BMC Bioinformatics. 2011 Dec 14;12(Suppl 14):S5. doi: 10.1186/1471-2105-12-S14-S5 (PMC3287470; doi:10.1186/1471-2105-12-S14-S5)
Supplement: Additional file 2 — The full list of predicted domains and motifs on CLEC17A. [file 1471-2105-12-S14-S5-S2.doc]

# Additional File 2

Full listing of predicted CLEC17A domains and motifs.

| **No** | **Type** | **Name** | **Position** | **Source** | **Description** |
| --- | --- | --- | --- | --- | --- |
| 1 | Region | Intracellular | 1 - 170 | TMHMM |  |
| 2 | Region | Transmembrane | 171 -193 | TMHMM |  |
| 3 | Region | Extracellular | 194 -378 | TMHMM |  |
| 4 | Domain | Alpha Helix | 166-183 188-215 218-224 226-241 275-284 295-303 | Jpred |  |
| 5 | Domain | Beta Sheet | 5-7 164-165 259-261 264-269 289-292 311-316 324-327 350-354 359-361 371-373 | Jpred |  |
| 6 | Domain | Alpha Helix | 78-80 173-185 193-214 217-244 275-284 295-304 | PSIPred |  |
| 7 | Domain | Beta Sheet | 4-5 123-124 164-165 170-172 189-192 259-261 264-269 288-290 311-316 324-326 350-353 368-374 | PSIPred |  |
| 8 | Domain | Lectin C-type domain | 271-374 | Pfam |  |
| 9 | Domain | C TYPE LECTIN 2 | 261-373 | Prosite | C-type lectin domain (Matrix) |
| 10 | Domain | C TYPE LECTIN 1 | 350-372 | Prosite | C-type lectin domain (Pattern) |
| 11 | Domain | PRO RICH | 31-153 | Prosite | Proline rich region |
| 12 | Domain | CK2 PHOSPHO SITE | 16-19 42-45 68-71 120-123 239-242 253-256 274-277 294-297 335-338 358-361 | Prosite | Casein kinase II phosphorylation site |
| 13 | Domain | PKC PHOSPHO SITE | 107-109 211-213 271-273 307-309 | Prosite | Protein kinase C phosphorylation site |
| 14 | Domain | ASN GLYCOSYLATION | 118-121 215-218 237-240 285-288 | Prosite | N-glycosylation site |
| 15 | Domain | MYRISTYL | 121-126 160-165 187-192 245-250 329-334 356-361 | Prosite | N-myristoylation site |
| 16 | Domain | CAMP PHOSPHO SITE | 250-253 374-377 | Prosite | cAMP-and cGMP-dependent protein kinase phosphorylation site |
| 17 | Motif | hemi-ITAM | 33-36 59-62 85-88 172-175 195-198 286-289 | Prosite | hemi-ITAM motif (YxxL) |
| 18 | Motif | EPN | 341-343 | Prosite | Speciﬁcity towards Mannose |
| 19 | Motif | WND | 359-361 | Prosite |  |
| 20 | Motif | CLV NDR NDR 1 | 250-252 | ELM | N-Arg dibasic convertase (nardilysine) cleav­age site (Xaa-|-Arg-Lys or Arg-|-Arg-Xaa) |
| 21 | Motif | CLV PCSK PC1ET2 1 | 167-169 | ELM | NEC1/NEC2 cleavage site (Lys-Arg-|-Xaa) |
| 22 | Motif | CLV PCSK SKI1 1 | 102-106 117-121 | ELM | Subtilisin/kexin isozyme-1 (SKI1) cleavage site ([RK]-X-[hydrophobic]-[LTKF]-|-X) |
| 23 | Motif | LIG CtBP | 121-125 | ELM | PxDLS motif that interacts with the CtBP protein |
| 24 | Motif | LIG CYCLIN 1 | 34-38 60-64 86-90 203-207 | ELM | Substrate recognition site that interacts with cyclin and thereby increases phosphorylation by cyclin/cdk complexes. Predicted protein should have the MOD CDK site. Also used by cyclin inhibitors. |
| 25 | Motif | LIG FHA 2 | 14-20 40-46 66-72 118-124 | ELM | Phosphothreonine motif binding a subset of FHA domains that have a preference for an acidic amino acid at the pT+3 position. |
| 26 | Motif | LIG MAPK 1 | 115-124 | ELM | MAPK interacting molecules (e.g. MAPKKs, substrates, phosphatases) carry docking mo­tif that help to regulate speciﬁc interaction in the MAPK cascade. The classic motif ap­proximates (R/K)xxxx#x# where # is a hy­drophobic residue. |
| 27 | Motif | LIG NRBOX | 242-248 | ELM | The nuclear receptor box motif (LXXLL) confers binding to nuclear receptors. |
| 28 | Motif | LIG PDZ 3 | 199-202 | ELM | Class III PDZ domains binding motif |
| 29 | Motif | LIG SH2 GRB2 | 26-29 52-55 78-81 | ELM | GRB2-like Src Homology 2 (SH2) domains binding motif. |
| 30 | Motif | LIG SH2 SRC | 26-29 52-55 78-81 | ELM | Src-family Src Homology 2 (SH2) domains binding motif. |
| 31 | Motif | LIG SH3 1 | 34-40 60-66 86-92 | ELM | This is the motif recognized by class I SH3 domains |
| 32 | Motif | LIG SH3 2 | 97-102 110-115 | ELM | This is the motif recognized by class II SH3 domains |
| 33 | Motif | LIG SH3 3 | 8-14 34-40 60-66 86-92 94-100 95-101 107-113 110-116 124-130 139-145 | ELM | This is the motif recognized by those SH3 do­mains with a non-canonical class I recognition speciﬁcity |
| 34 | Motif | LIG TRAF2 1 | 16-19 42-45 68-71 | ELM | Major TRAF2-binding consensus motif. Members of the tumor necrosis factor recep­tor (TNFR) superfamily initiate intracellular signaling by recruiting the C-domain of the TNFR-associated factors (TRAFs) through their cytoplasmic tails. |
| 35 | Motif | LIG USP7 1 | 92-96 | ELM | The USP7 NTD domain binding motif vari­ant based on the MDM2 and P53 interac­tions. |
| 36 | Motif | LIG WW 2 | 110-113 | ELM | PPLP is the motif recognized by WW do­mains of Group II |
| 37 | Motif | LIG WW 3 | 96-100 99-103 | ELM | WW domain of group III binding motif |
| 38 | Motif | LIG WW 4 | 27-32 53-58 79-84 138-143 147-152 | ELM | Class IV WW domains interaction motif; phosphorylation-dependent interaction. |
| 39 | Motif | MOD CK2 1 | 13-19 39-45 65-71 236-242 | ELM | CK2 phosphorylation site |
| 40 | Motif | MOD GlcNHglycan | 94-97 158-161 | ELM | Glycosaminoglycan attachment site |
| 41 | Motif | MOD GSK3 1 | 143-150 | ELM | GSK3 phosphorylation recognition site |
| 42 | Motif | MOD N-GLC 1 | 27-32 53-58 79-84 117-122 214-219 236-241 | ELM | Generic motif for N-glycosylation. Shakin-Eshleman et al. showed that Trp, Asp, and Glu are uncommon before the Ser/Thr po­sition. Eﬃcient glycosylation usually occurs when 60 residues or more separate the gly­cosylation acceptor site from the C-terminus |
| 43 | Motif | MOD PKB 1 | 231-239 | ELM | PKB Phosphorylation site |
| 44 | Motif | MOD ProDKin 1 | 27-33 53-59 79-85 138-144 147-153 | ELM | Proline-Directed Kinase (e.g. MAPK) phos­phorylation site in higher eukaryotes. |
| 45 | Motif | TRG ENDOCYTIC 2 | 33-36 59-62 85-88 195-198 | ELM | Tyrosine-based sorting signal responsible for the interaction with mu subunit of AP (Adap­tor Protein) complex |
| 46 | Motif | TRG LysEnd APsAcLL 1 | 242-247 | ELM | Sorting and internalisation signal found in the cytoplasmic juxta-membrane region of type I transmembrane proteins. Targets them from the Trans Golgi Network to the lysosomal-endosomal-melanosomal compart­ments. Interacts with adaptor protein (AP) complexes |
| 47 | Modiﬁcation | N-Glycosylation | 118-118 215-215 237-237 | NetNGlyc |  |
| 48 | Modification | O-Glycosylation | 30-30 42-42 56-56 68-68 81-81 82-82 94-94 95-95 107-107 | NetOGlyc |  |
