# Supplementary material for: Integrative analysis workflow for the structural and functional classification of C-type lectins
Source: BMC Bioinformatics. 2011 Dec 14;12(Suppl 14):S5. doi: 10.1186/1471-2105-12-S14-S5 (PMC3287470; doi:10.1186/1471-2105-12-S14-S5)
Supplement: Additional file 3 — Additional background, as well as materials and methods for the experimental investigation of predicted N-glycosylation sites. [file 1471-2105-12-S14-S5-S3.doc]

# Additional File 3

## Background

Here we give some of the background materials on the different levels of analyses for predicting the functions and interactions of novel C-type lectins.

**Predicting C-type Lectin Structure and Functions**

One of the basic approaches to determining putative functions of a novel C-type lectin is to analyze its amino acid sequence. It is generally accepted that protein functions can be ‘*inherited through homology’*, that is, proteins with similar sequences tend to share similar functions. The homology among the protein sequences need not be contiguous and can be restricted to small, independent sub-regions, i.e. protein domains. Thus, with the advent of computational methods and databases to store such domains and their associated functions, e.g Pfam, PROSITE and SMART, it becomes evident that the first steps to analyze a C-type lectin with unknown functions is to perform a sequence-based analysis on its amino acid sequence to determine its (i) molecular function, (ii) biological process, and (iii) cellular location. This is followed by a structure-based analysis to further understand their features and interaction mechanisms on the atomic level.

***Domains and Motifs in C-type Lectins***

Protein domains are defined as regions in the protein sequence that either have associated functions, or specific folds/structures. Their identification and classification can be made according to sequence or structural similarities. By locating the presence of such domains along the sequence of a novel C-type lectin, one could possibly infer its functions and physiological roles. A protein is classified as a C-type lectin due to the presence of C-type CRDs in its sequence. Although it has been shown that the CRD alone may not guarantee carbohydrate binding, it has nonetheless been used to identify new C-type lectins, until experimentally confirmed otherwise.

Within the CRD, there are variations that may make the C-type lectin function differently. For instance, presence of a particular triplet of amino acid residues along the loop region in the CRD, commonly referred to as the EPN motif, indicates the C-type lectin’s specificity towards mannose. Similarly, two other motifs and their functions have been identified in the CRD of other C-type lectins, i.e. the QPD motif for galactose specificity and the WND motif for coordinating the binding of Ca^2+^ ions to the CRD binding site.

Aside from the CRD, the presence of a transmembrane region and its location along the sequence, as well as the protein orientation are also relevant to analyze those C- type lectins that function as membrane receptors. C-type lectins can be loosely classified as Type I or Type II, depending on their membrane orientations. Type I C-type lectins have their N-termini in the extracellular region, and typically have more than one CRDs along their sequences, while Type II C-type lectins are oriented in the opposite direction, and have only one CRD. Other C-type lectins, such as collectins, exist as soluble proteins, recognizing and binding to carbohydrate moieties found on the cell surfaces of pathogens. Upon binding the carbohydrate molecules, the collectins will associate themselves with other cell surface receptors, triggering phagocytosis or oxidative killing of the pathogens. Hence, it is possible to postulate the ‘mode of operation’ of a C-type lectin by determining whether it is membrane-bound or secreted.

One of the primary functions of C-type lectins is the recognition of carbohydrates. Upon binding the ligands, a series of events can be triggered, resulting in the internalization of pathogens for degradation or antigen presentation. Some C-type lectins can also activate intracellular signaling. For instance, Dectin-1 has a hemi-ITAM motif in the intracellular region, such that upon binding an external ligand, the motif will be phosphorylated, leading to the recruitment of the protein tyrosine kinase Syk. This initiates a signaling cascade that results in the secretion of interleukins IL-2, IL-10 and IL-12, and subsequently T- cell maturation. Although the molecular mechanisms are still unclear, it is believed that there are cross-talk interactions between the Toll-like receptor (TLR) signaling pathways and the pathways activated by C-type lectins. All these processes are mediated by the presence of other domains and motifs not found in the CRD. Hence searching the sequences of novel C-type lectins for such features could shed some light on their intracellular activity.

***Molecular Modeling and Docking***

The functions and potential interacting partners of novel C-type lectins can also be discovered by modeling and analyzing their molecular structures. Structure-based function prediction has received considerable interest in the recent years.

A major challenge for structure-based analysis is to create the structural model of the C-type lectins. In general, there are two approaches for this task - (i) comparative modeling, which includes homology modeling and threading, and (ii) de novo structure prediction. Given current developments, the former approach is more viable. However, its applicability depends on the presence of homologous sequences in structural databases such as the Protein Data Bank (PDB). For C-type lectins, several CRD structures are already elucidated and deposited into the PDB database. Thus, given the highly conserved nature of the CRD, it is possible to produce the molecular structures of the corresponding regions on other C-type lectins for further analyses.

Upon building the molecular model of the C-type lectins, their structural features could be studied in more detail. In particular, the search for putative binding sites on the protein surface is an important task as it provides clues to the functions of the C-type lectins, and also helps reduce the computational time required for subsequent docking studies and virtual screening. A typical approach for locating binding sites involves searching the protein surface for cavities, either through their geometric properties, or by means of energy-based probing methods.

Following binding site detection, we can assess whether the C-type lectins interact with certain ligands (and thereby determining their physiological roles). An obvious class of such ligands is carbohydrates, or glycans. They are sugars (monosaccharides or polysaccharides) and can be found conjugated to other macromolecules, especially secreted and cell-surface proteins, in all living things. By identifying the interacting glycans and their binding mechanisms, not only can we discover their functions, molecules can be engineered so that they target specific C-type lectins in applications such as cell based cancer therapy. This process can also be scaled up by building in silico libraries of ligand models for rapid assessment of their docking capabilities, i.e. virtual screening.

**Experimental Investigation of Predicted N-Glycosylation Sites**

From the workflow, it is predicted that CLEC17A is glycosylated at 3 amino acid positions, i.e. 118, 215, and 237. However, N-linked glycosylation sites are usually found on secreted proteins, or the extracellular regions of membrane bound ones. Since position 118 is found to be within the cytoplasmic region of CLEC17A, we deemed it as not a likely site and excluded that position from further consideration.

To determine the plausibility of the other two sites, CHO-K1 cells were transfected with N-terminus enhanced green fluorescent protein (EGFP)-tagged full-length human CLEC17A gene. In addition, four EGFP-tagged truncated mutants were generated and transfected to the same cell line for expression. These segments were truncated from the full length CLEC17A protein at amino acid positions 119, 171, 194, and 216 (Figure 1A). Subsequently, the transfected cells were lysed, the lysates were separated using electrophoresis, and the proteins of interest were probed using the primary EGFP monoclonal mouse antibody (JL-8, Clontech) and the secondary anti-mouse HRP-conjugated antibody (Promega).

To assess the presence of N-linked glycosylated sites, a separate set of protein samples were treated with N-glycanase (PNGase) before electrophoresis. N-glycanase is a peptide that releases intact N-linked oligosaccharides from the glycoproteins, which in this case, are the truncated segments of CLEC17A. If the protein segment is N-glycosylated, treatment with N-glycanase will remove the glycans, thus reducing the molecular weight of the segment. The theoretical molecular weight of the full length CLEC17A, and its different segments are summarized in Figure 1A.

**Materials and Method
*Cell Transfection Process***

To affirm the presence of N-Glycosylation sites, 4 mutants of the human CLEC17A gene were generated, each being a segment of the full length CLEC17A protein truncated from amino acid positions 119, 171, 194 and 216 respectively (Figure 1A). The full-length protein and the segments were tagged with enhanced green fluorescent protein (EGFP) at their N-terminus and transfected into separate CHO-K1 cells. Subsequently, the transfected cells were lysed with Mammalian Protein Extraction Reagent (M-PER) (Pierce) for analysis.

***Electrophoresis***

Protein samples (13 μl) of the cell lysates from the full length CLEC17A and the four truncated variants were added to the loading buffer (5 μl) and spun down. When sample reduction is required, reducing buffer (2 μl) is added and the sample is heated to 95^o^C for 5 minutes.

***N-Glycanase Treatment***

Protein samples treated with N-glycanase (Peptide-N-Glycosidase F) (Prozyme) were added to the reaction buffer (3.375 μl). They were then added to the denaturation solution (0.9375 μl, 2% SDS, 1M β-mercaptoethanol) (Prozyme) and subjected to denaturation by heating at 100^o^C for 5 minutes. The mixture was then allowed to cool and the detergent solution (0.9375 μl, 15% NP-40 solution) (Prozyme) was added. The mixture was incubated for 2 hours to overnight at 37^o^C with N-glycanase (0.5 μl). The protein samples were then loaded into Nu-Page 4-12% Bis Tris gels. The protein molecular weight standard used for the ladder was MagicMark XP Western Protein Standards (Invitrogen).

***Electrotransfer***

After electrophoresis, the protein samples were transferred from the gel to Polyvinylidene fluoride (PVDF) membranes (Immobilon-P) (Millipore) and probed with 1:2000 dilution of EGFP monoclonal mouse antibody (JL-8) (Clontech) as the primary antibody and 1:4000 dilution of anti-mouse HRP-conjugated antibody (Promega) as the secondary antibody. The protein samples on the membrane were detected using enhanced chemiluminescence plus (ECL plus) (Amersham Bioscience) following the protocol from the manufacturer.

##
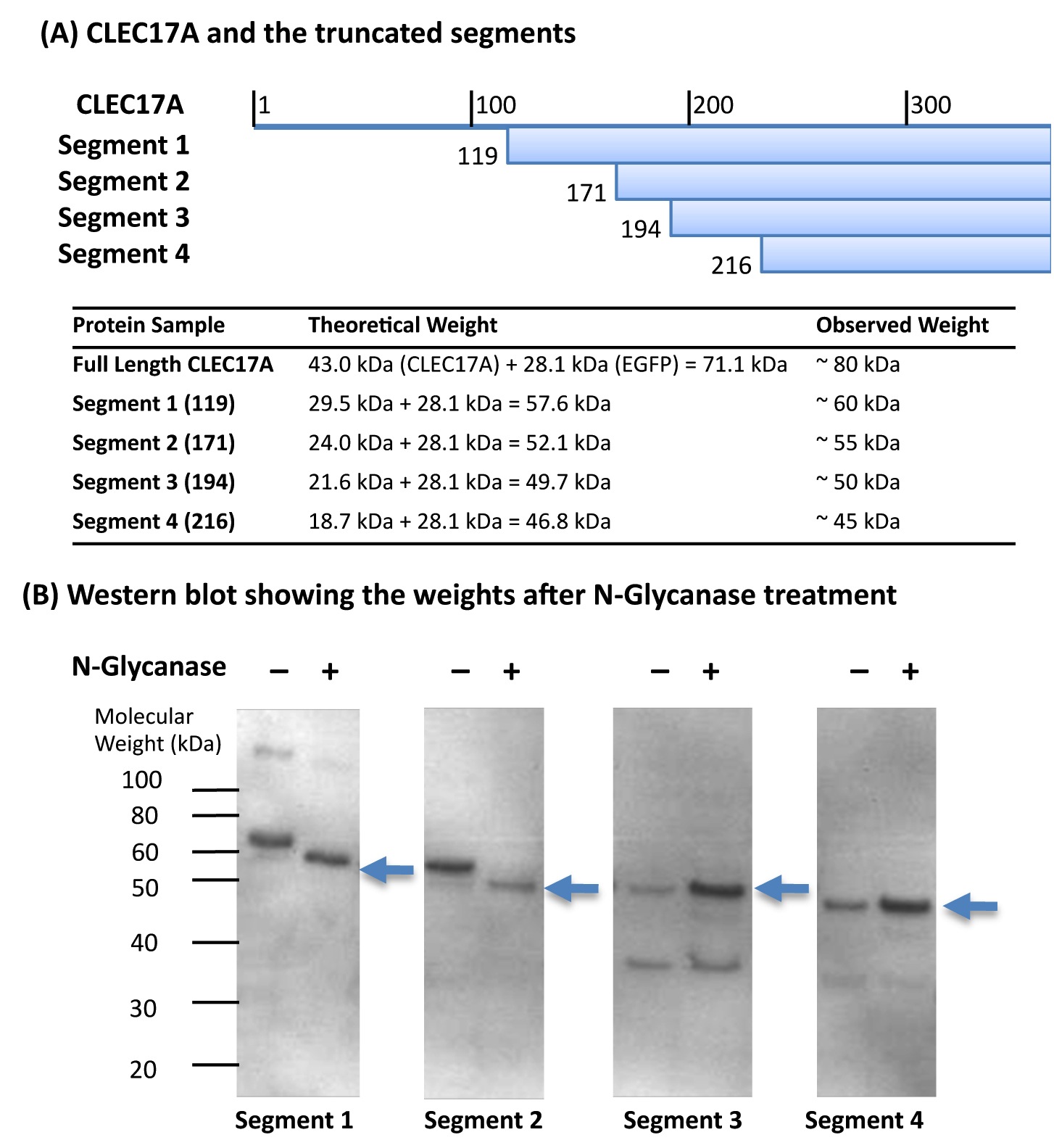


## Figure 1 - Experimental investigation of the N-glycosylation sites on CLEC17A

(A) The truncated segments and their theoretical and observed molecular weights. (B) Western blot images showing the weights of the individual segments upon treatment with N-glycanase. Segments 1 and 2 show observable reduction in their molecular weights, while the weights of segments 3 and 4 remain the same. This can be explained by the lack of the transmembrane region in segments 3 and 4, which is located between 171 and 193.

**Results**

Figure 1B shows the western blot of the individual segments with and without N-glycanase treatment. As expected, there is an observed reduction in molecular weight for segments 119 and 171. Since this weight reduction is almost similar, it indicates that the glycosylated sites lie beyond 171. To recap, the N-glycosylated sites in the extracellular region of CLEC17A are predicted to be at positions 215 and 237. Interestingly, segments 194 and 216 showed no significant reduction in weight. However, by taking into account the transmembrane region is predicted to be between 171 and 193, it becomes clear that segments 194 and 216 are not glycosylated because they lack the transmembrane signal to enter the endoplasmic reticulum where N-glycosylation is initiated. Taking both experimental and predicted features into consideration, it is very likely that the predicted transmembrane region and N-linked glycosylation sites are correct.
